# Phosphate intake, hyperphosphatemia, and kidney function

**DOI:** 10.1007/s00424-022-02691-x

**Published:** 2022-05-05

**Authors:** Isabel Rubio-Aliaga, Reto Krapf

**Affiliations:** 1 Institute of Physiology, National Center of Competence in Research NCCR Kidney.CH, University of Zurich, Winterthurerstrasse 190, 8057 Zurich, Switzerland; 2 Synlab Suisse, 6002 Lucerne, Switzerland; 3 Department of Medicine, University of Basel, 4056 Basel, Switzerland

**Keywords:** Dietary phosphate, Hyperphosphatemia, Kidney function, Chronic kidney disease, Cardiovascular disease, Mortality risk

## Abstract

Phosphate is essential in living organisms and its blood levels are regulated by a complex network involving the kidneys, intestine, parathyroid glands, and the skeleton. The crosstalk between these organs is executed primarily by three hormones, calcitriol, parathyroid hormone, and fibroblast growth factor 23. Largely due to a higher intake of ultraprocessed foods, dietary phosphate intake has increased in the last decades. The average intake is now about twice the recommended dietary allowance. Studies investigating the side effect of chronic high dietary phosphate intake suffer from incomplete dietary phosphate assessment and, therefore, often make data interpretation difficult. Renal excretion is quickly adapted to acute and chronic phosphate intake. However, at the high ends of dietary intake, renal adaptation, even in pre-existing normal kidney function, apparently is not perfect. Experimental intervention studies suggest that chronic excess of dietary phosphate can result in sustained higher blood phosphate leading to hyperphosphatemia. Evidence exists that the price of the homeostatic response (phosphaturia in response to phosphate loading/hyperphosphatemia) is an increased risk for declining kidney function, partly due by intraluminal/tubular calcium phosphate particles that provoke renal inflammation. High dietary phosphate intake and hyperphosphatemia are progression factors for declining kidney function and are associated with higher cardiovascular disease and mortality risk. This is best established for pre-existing chronic kidney disease, but epidemiological and experimental data strongly suggest that this holds true for subjects with normal renal function as well. Here, we review the latest advances in phosphate intake and kidney function decline.

## Introduction

Phosphate is essential in living organisms. In nature, phosphorous is commonly found in combination with oxygen as phosphate (PO_4_^3−^). In humans, phosphate has a wide range of functions. Together with calcium, it forms hydroxyapatite in the skeleton, is part of the energy carrier molecule adenosine triphosphate and the backbone of RNA and DNA molecules, and is present in cell membranes as phospholipids. Phosphorylation of proteins plays a major role in intracellular signalling and with its buffering capacity phosphate contributes to acid–base homeostasis. Phosphate is predominantly an intracellular anion. The ratio of intra- to extracellular concentration is 10 to 15:1. Most phosphate (85%) is found in bone tissue and 14% in soft tissue, and a very small fraction is found in the extracellular space [81]. Intracellular phosphate is found in organic and inorganic forms, while in extracellular fluids, it is present mostly (85%) in the inorganic form as free ions or complexed to cations such as calcium and magnesium and 15% of the phosphate is bound to proteins. The inorganic fraction present in the extracellular space and blood plays a crucial role in phosphate homeostasis despite constituting only a small proportion (< 0.1%) of total body phosphate content. In the organism, a complex network that involves the skeleton, the kidneys, the parathyroid glands, and the intestine regulates phosphate levels [42]. The crosstalk between the organs is mainly guaranteed by the action of active vitamin D (calcitriol), parathyroid hormone (PTH), fibroblast growth factor 23 (FGF23), and αKlotho. Phosphate levels in plasma are maintained in a broader range than the levels of the related mineral calcium and vary between 0.8 and 1.5 mM [81]. Deviations from this range increase the risk of disease. Chronic hypophosphatemia causes rickets in children and osteomalacia in adults and is associated with cardiomyopathy, whereas chronic hyperphosphatemia has long been known to cause soft tissue calcification. Hyperphosphatemia and phosphate levels at the higher end of the normal range have been associated with a risk of cardiovascular events, coronary calcification, mortality, and diminished kidney function [19, 108]. As phosphate is present in most foods and renal phosphate conservation in states of low dietary intake is extremely efficient, hypophosphatemia rarely has a dietary or external cause except for the refeeding syndrome [65]. The main topics of this review are the evidence for chronic high phosphate diet leading to hyperphosphatemia despite normal renal function, and for high dietary phosphate intake to be deleterious for renal function.

## Dietary sources of phosphate

**Fig. 1:**
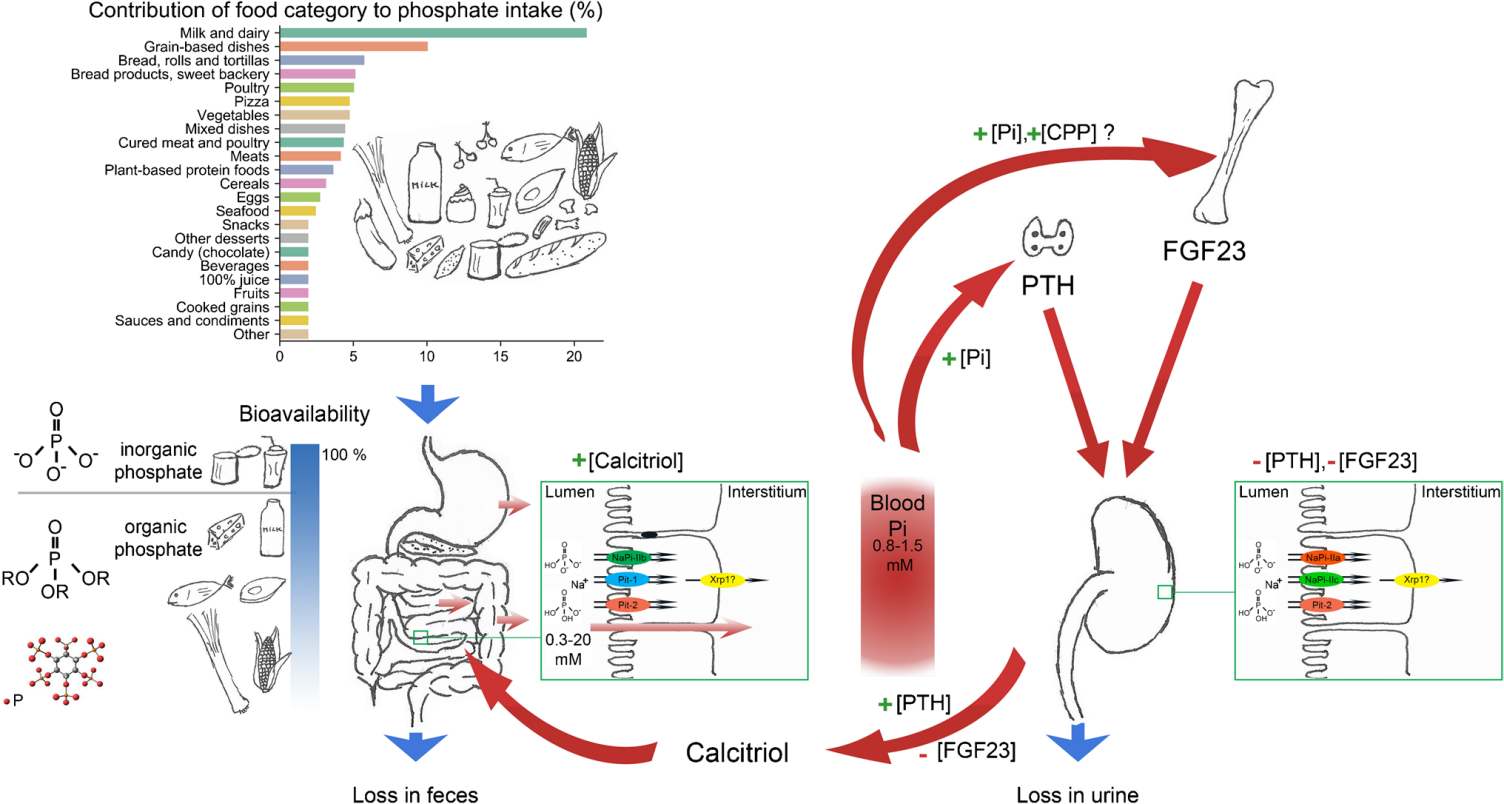
Dietary phosphate and phosphate homeostasis in humans. Milk and dairy products, followed by grain-based dishes and bread, are the major contributors of dietary phosphate, when food categories are considered [14]. Phosphate in the diet has different bioavailability depending on the chemical structure and source. Inorganic phosphate from ultrapocessed foods such as beverages and canned food has a high bioavailability followed by organic phosphate from animal origin. Phosphate in plants is mostly present as phytates and has the lowest bioavailability [108]. When phosphate enters the gastrointestinal tract, phosphate permeability occurs already in the stomach as assessed in intestinal cell models [55]. This paracellular transport occurs along the whole intestine. The active sodium-dependent transports occur predominantly in the small intestine, and NaPi-IIb is probably the most predominant phosphate transporter and it is regulated by calcitriol, an hormone secreted by the kidneys [66]. The mechanisms how phosphate leaves the enterocytes are still unclear, but several studies suggest it may be mediated by Xpr1, although the basolateral localization has not been confirmed yet [38]. Phosphate is maintained in the blood at concentrations around 0.8 to 1.5 mM. The kidneys play the major role in excreting excess phosphate from the diet [38]. When phosphate levels in plasma raise, PTH and FGF23 secreted from parathyroid glands and osteocytes, respectively, decrease the expression and translocation especially of NaPi-IIa and NaPi-IIc to the apical membrane which results in lower reabsorption and higher phosphate excretion in the urine. These sodium-dependent phosphate transporters are localized in the proximal cells in the kidney [38]. In these cells, Xpr1 may also mediate efflux from the epithelial cells. High FGF23 concentrations inhibit calcitriol synthesis, whereas high PTH concentrations in the blood promote calcitriol synthesis in the kidney [10]

Phosphate is widely distributed in food as inorganic and organic phosphate. Protein foods (fish and meat) have the highest content of phosphate per portion, followed by dairy products such as yogurt and milk, nuts, corn, vegetables, and legumes that have similar amounts of phosphate. The lowest content is found in beverages such as beer and colas [101]. However, these beverages are often consumed in considerable volumes and, therefore, conceivably constitute in such instances an important acute phosphate load. Considering the average daily diet composition, dairy products are the major contributors to phosphate intake followed by grain-based dishes and bread products (Fig. 1) [14]. Not only phosphate content in food but also its bioavailability determines the amount of phosphate absorbed by the organism. Inorganic phosphate has a higher bioavailability than organic phosphate, which hast to be first hydrolysed in the intestine by phosphatases in order to be absorbed [102]. Furthermore, inorganic phosphate in beverages is more rapidly absorbed than inorganic phosphate in foods. Organic phosphate of animal origin has a higher bioavailability than phosphate of plant origin, as the intestinal phosphatases in the organism are not able to hydrolyse the organic phosphate, mostly phytates, found in plants. However, fermentation, acidification, cooking, and constituents of the gut microbiota, which are phytase sources, allow that around 30% of the organic phosphate present in plants is liberated and absorbed in the human intestine [93, 108]. In addition, the dietary ratio of phosphate to other minerals, such as calcium and magnesium, also affects phosphate bioavailability. Increased dietary content of these divalent cations decreases the bioavailability of dietary phosphate due to the intestinal formation of poorly soluble mineral complexes [81].

Phosphate is also present in ultraprocessed food (UPF). Phosphate salts are use in food manufacturing for retention of smoothness, binding, and moisture [81]. Nearly half of the top-selling foods in groceries contain phosphate additives [60]. The amount of phosphate added as preservative or additive seem to vary depending on the food category, being lower in soups, canned vegetables, and bread and higher in dry food mixes, dairy products, and cereals. Moreover, the kind of phosphate added depends also on the food category [14]. Of note, the amount of phosphate additives in the diet is not negligible when UPF are consumed routinely and may increment phosphate consumption to as much as 1 g/day [9, 60]. Moreover, phosphate salts added as additives are inorganic phosphate with an almost 100% bioavailability (Fig. 1), indicating that independently of the amount present in the UPF, the phosphate will almost completely be absorbed in the intestine [14]. In the last decades, consumption of UPF has increased [74]. UPF consumption represents 20 to almost 60% of the total energy intake consumed depending on the different population groups studied [16, 63, 68, 69, 78, 95]. Higher UPF consumption is associated with higher BMI, lower income, and younger age [53, 95], and with higher consumption of sugars, sodium, and fats, but lower consumption of potassium, iron, and vitamins [69, 100]. Of note, when phosphate intake was estimated in these studies, it was lower in the population groups consuming more UPF, indicating underestimation of phosphate consumption by the conventional food assessment methods [69, 78, 83].

## Dietary phosphate intake

Assessment of phosphate intake is difficult, not only due to the limitations of the methods but also because food companies are not required to label and report phosphate-containing additives. Underestimation of phosphate intake is mainly due to UPF consumption and ranks between 14 and 40% of the phosphate reported [15, 83]. Most studies rely on (single) 24-h recall or food frequency questionaries to assess phosphate intake. Data on phosphate intake in the general population have been obtained in different countries. There is a relatively low cross-cultural variation in dietary phosphate intake: in the USA, the average phosphate intake in different cohorts varies between 1100 and 1400 mg/day [3, 20, 32, 79], with individual intake varying between 280 and 3000 mg of dietary phosphate per day [71], with lower intake in females than males [14]. In a Finnish cohort, an average of 1600 mg/day phosphate intake was reported [46]. In a Korean cohort, values of 800 mg/day were reported [58]. In Iran, an average intake of phosphate of 1200 mg/day was assessed, and, in another cohort, individuals reported between 180 and 1100 mg of phosphate intake per day [6, 70]. Additionally, in a Chinese cohort, a phosphate intake between 700 and 1400 mg/day was assessed [114]. Taken together, these data and considering the reported underestimation, we can estimate than on average, an adult consumes around 1500 mg of phosphate per day. Considering that the US-recommended dietary allowance (RDA) of phosphate for adults is 700 mg/day [1], concern has arisen that the current mean intake of about twice RDA phosphate consumption may induce systemic changes and be harmful for health [29, 89]. The RDA establishes the amount of an essential nutrient required to maintain the nutrients needs, yet the acceptable daily intake (ADI) is defined as an estimate of the amount of a food additive (expressed on a body weight basis) that can be ingested on a daily basis over a lifetime without appreciable risk to health. Recently, the European Food and Safety Administration (EFSA) has proposed, based on older studies performed in animals, an acceptable ADI of phosphate of 40 mg/kg/day, i.e. for a 70 kg person a consumption of 2800 mg of phosphate per day [85]. This ADI suggests that most of the general population consumes amounts of phosphate below the risk of toxicity, yet for certain population groups, such as CKD patients and probably infants, the ADI is probably lower [15, 53, 89].

## Phosphate homeostasis

### Phosphate absorption

On average, 1500 mg of dietary phosphate reaches daily the gastrointestinal tract. After a meal, intestinal luminal phosphate concentrations vary between 0.3 and 20 mM in human [24], rat [54, 67], and mouse [44, 55], with higher values in the proximal than in the distal small intestine [44] (Fig. 1). Depending on the type of diet, between 40 and 60% of the dietary phosphate content is absorbed in the intestine [40]. Two mechanisms are responsible for this absorption: transcellular sodium-dependent active transport and paracellular transport. Under normal dietary conditions, NaPi-IIb (SLC34A2) is the main sodium-dependent phosphate transporter in the intestine [66]. NaPi-IIb activity is mainly regulated by low dietary phosphate and calcitriol, but also by other factors such as estrogens, glucocorticoids, and epidermal growth factor (EGF) [40]. Two SLC20 family members are also expressed in the intestine, Pit-1 (SLC20A1) and Pit-2 (SLC20A2), but their contribution to active phosphate transport is probably lower than 10% [66]. Pit-2 seems to play a role when dietary phosphate intake is restricted and, therefore, could contribute to preventing phosphate depletion [87]. Recently, phosphate uptake studies in intestinal brush border membranes from rats suggest that Pit-1 may play a role in chronic kidney disease (CKD) [43]. The mechanism by which phosphate leaves the enterocytes at the basolateral side and reaches the portal vein is still unclear. Recently, XPR1, an entry receptor of a murie gamma retrovirus (X-MLV) was suggested as phosphate exporter [33]. In vitro studies showed that Xpr1 allows phosphate efflux, and may be responsible for the intestinal (and renal) basolateral transport, although its cellular localization needs further clarification [38] (Fig. 1).

The active transport is fundamental at luminal phosphate concentrations below 2 mM, but the paracellular pathway plays the major role in phosphate absorption under normal physiological conditions [24, 40, 66]. Dilution potential measurements and radiolabelled flux experiments in mice revealed that the tight junctions along the whole gastrointestinal tract are highly permeable to phosphate with a slight preference for monovalent phosphate [55]. The amount of passively absorbed phosphate via the paracellular route is not affected by ablation of intestinal NaPi-IIb and it is not regulated by calcitriol [39, 55]. This lack of regulation of the intestinal paracellular phosphate transport is especially important in CKD patients. At an average dietary intake, phosphate absorption rate is similar in healthy volunteers and CKD patients despite the lower calcitriol levels in these patients, as shown by radiotracer studies [103]. Luminal-to-blood phosphate gradients and transepithelial potential differences govern the phosphate paracellular transport. Studies defining the molecular identity of the proteins involved in phosphate permeability at the tight junctions are under way. Claudin 3 is a candidate protein. Lithocholoic acid increases intestinal phosphate absorption by reducing Claudin 3 expression. Surprisingly, although the passive route is not regulated by calcitriol, the regulation of Claudin 3 function by lithocholic acid occurs in a Vitamin D Receptor (Vdr)-dependent manner [37]. Identifying activators of this sealing claudin may lead to drug targets in CKD patients, as inhibiting phosphate intestinal absorption is central to prevent phosphate overload/hyperphosphatemia in this population [40, 61, 66].

### Phosphate reabsorption

Current knowledge indicates that under normal kidney function, the kidneys and not the intestine play the main role in maintaining phosphate homeostasis, eliminating excess phosphate from dietary intake and metabolism in the urine [66]. NaPi-IIa (SLC34A1) and NaPi-IIc (SLC34A3) are the active sodium-dependent phosphate transporters expressed solely in the apical membrane of renal proximal epithelial cells [38]. Regulation of these transporters ensures maintenance of phosphate homeostasis. Once phosphate is absorbed in the organism, matching renal phosphate reabsorption to phosphate intestinal absorption becomes essential. In cases of dietary phosphate restriction, NaPi-IIa and NaPi-IIc expression are increased (in chronic adaptation) and the transporters are translocated to the apical membrane (in acute and chronic adaptation) to allow increased reabsorption. Though when a high phosphate diet is ingested, the opposite occurs [62, 90, 109]. PTH and FGF23 are the major negative regulators (Fig. 1), while dopamine is the major positive regulator of these transporters’ expression. When an acute high phosphate load is imposed either orally or intravenously, PTH plays the predominant role increasing its blood levels and in parallel to the phosphaturia provoked [80, 94, 106]. PTH and FGF23 have opposing effects on net calcitriol production in the kidney by modulating the expression of the key enzymes in calcitriol synthesis and inactivation, CYP27B1 and CYP24A1 [10]. High PTH levels enhance calcitriol production, whereas high FGF23 levels diminish calcitriol production. FGF23 requires the presence of its obligatory co-receptor αKlotho for its action in the kidney [42]. NaPi-IIb, Pit-1, and Pit-2 are also expressed in the nephron, but their contribution to phosphate reabsorption is probably minor [61, 77]. At the basolateral side of renal cells, Xrp1 is a promising candidate for basolateral efflux (Fig. 1). Renal ablation of Xrp1 function leads to hypophosphatemia in mice and a Fanconi-like syndrome [4]. More detailed studies are necessary to delineate the quantitative role of this transporter and the mechanisms by which it is regulated.

### Phosphate turnover

NaPi-IIb, Pit-1, and Pit-2 are also expressed in extra renal and extra intestinal tissues allowing the continuous exchange of phosphate between the organs and the extracellular space, i.e. they are involved in transcellular phosphate balance [38]. Phosphate is present in all cells, but the skeleton is the organ containing the major proportion of total body phosphate (85%). PTH and calcitriol are the main hormones regulating phosphate turnover in the bone. Other hormones such as IGF1 and osteopontin (OPN) are also regulated by phosphate concentrations and implicated in bone homeostasis [18].

### Phosphate sensing

The mechanisms by which the organism senses variations in phosphate concentration [8, 18] are still incompletely understood. Although it was initially proposed that the intestine may play a role in phosphate sensing by assessing the phosphate load and sending signals to the kidney to regulate phosphate excretion, there is no evidence for a gut-derived phosphaturic molecule [59, 94]. The Pit-1/Pit-2 heterodimer complex senses variations in extracellular phosphate concentrations leading to the regulation of FGF23 secretion in bone [11, 12]. Following a phosphate load, ablation of Fgfr1c function in bone leads to impaired FGF23 secretion by modulating Galnt3 activity, whereas activation leads to enhanced FGF23 secretion [104, 105]. Other studies suggest, however, that it may not be the ambient phosphate levels per se but the levels of colloidal protein-mineral complexes specifically calciprotein particles (CPP) that trigger FGF23 secretion in bone after a dietary phosphate load [2] (Fig. 1). On the other hand, phosphate is a non-competitive antagonist of the calcium sensing receptor (CaSR). Increased phosphate levels lead, both in vivo and in vitro, to higher PTH secretion from the parathyroid glands via the CaSR [17]. IP6Ks may be phosphate sensors and regulators not only in yeast and plants but also in mammals [76]. Intracellular phosphate changes are sensed by inositol hexakisphosphate kinases 1 and 2 (IP6K1 and 2) modulating IP7 synthesis which regulates Xpr1 activity [18, 112]. Moreover, administration of an IP6Ks inhibitor (SC-1919) in rats and monkeys leads to decrease in phosphate plasma levels [76]. Targeting any of these putative sensors could help controlling hyperphosphatemia in CKD, although side effects may be relevant due to the ubiquitous expressions and pleiotropic function of these proteins.

## Impact of dietary phosphate on phosphate homeostasis

A change to a high phosphate diet induces rapid renal elimination of the excess phosphate. Moreover, acute phosphate intake leads to peaks of hyperphosphatemia. In rats fed a low phosphate diet, a phosphate bolus led to a dramatic increase of phosphate levels in plasma reaching values above 5 mM [34]. This increase was less dramatic but also clearly appreciable in mice fed a standard phosphate diet [59]. Recurrent hyperphosphatemic peaks provoked, for example, by ingestion of food and beverages with rapidly absorbed inorganic phosphate may be deleterious for the organism. Further studies are needed to investigate this hypothesis.

However, whether a chronic consumption of a high phosphate diet under normal kidney function leads to a sustained increase in the phosphate levels in the blood and modulation of the hormones involved in phosphate homeostasis is still not entirely clear. As mentioned before, phosphate dietary assessment is difficult. Therefore, it is not surprising that epidemiological studies are not uniformly consistent in finding a positive correlation between dietary phosphate intake and phosphate levels in the blood [25, 31]. Moreover, consumption of specific food categories such as milk and dairy products with and without added inorganic phosphate or noodles with added inorganic phosphate has been associated with higher serum phosphate levels [75, 92]. These results must be interpreted with caution due to the limitations of phosphate dietary assessments methods, lack of parallel phosphate determination in urine and stool, and incompleteness of food databases [15]. In addition, the time of sample taken is crucial for phosphate levels determination, as phosphate follows a circadian rhythm, which further complicates the interpretation of the clinical and dietary data obtained [108].

Intervention studies have better controlled conditions that help interpreting the data, although long exposures are difficult to perform. In an intervention study, 20 young healthy adults were subjected for 6 weeks to either a high inorganic phosphate diet (regular plus supplemented with 0.55 mM neutral sodium phosphate/kg body weight) or a low phosphate (regular diet plus phosphate binders). After 6 weeks, phosphate in the blood increased significantly in the group ingesting the high phosphate diet. FGF23, PTH, and αKlotho were also increased, whereas calcitriol tended to decrease. In the low phosphate group, no changes were observed in any parameter [73]. In a further study with young adults, after 4-day adaptation and 5-day treatment with 500 mg phosphate binders or 2500 mg phosphate diet, the individuals consuming a higher phosphate diet had increased blood phosphate, PTH, and FGF23 concentrations when compared to the individuals consuming the low phosphate diet, yet when compared to baseline, there was no increase of the blood phosphate levels. A clear increase in urinary fractional excretion of phosphate was observed [13]. However, the finding that a higher phosphate load induces hyperphosphatemia in subjects with normal renal function is not uniform in the literature. Thirteen healthy young men underwent a 4-week intervention study with 9-day period of control (1500 mg phosphate/day), supplemented (2300 mg phosphate/day), and restricted (625 mg phosphate/day). This small study did not detect significant changes in serum phosphate under the high phosphate diet as well as in PTH, calcitriol, and FGF23 levels. However, during restricted phosphate intake, decreased phosphate, FGF23, and PTH levels and increased calcitriol serum concentrations were detected [5]. It is conceivable that the fractional absorption rate of phosphate was different in these studies, but no direct comparison can be made, as no stool data are available.

In a recent study, mice aged 6 months were fed over 1 year with a high phosphate diet that mimic the reported intake in the human population, i.e. twofold the standard phosphate diet [107]. Compared to the mice fed a standard phosphate diet, mice on a high phosphate diet developed hyperphosphatemia (on average 0.5 mM (26%) higher), clearly higher PTH levels and a tendency to higher FGF23 levels. In view of the hyperphosphatemia, calcitriol inappropriately increased. As PTH increased more than the relative increase in FGF-23, secondary hyperparathyroidism may have caused an increase in calcitriol in this model.

**Fig. 2:**
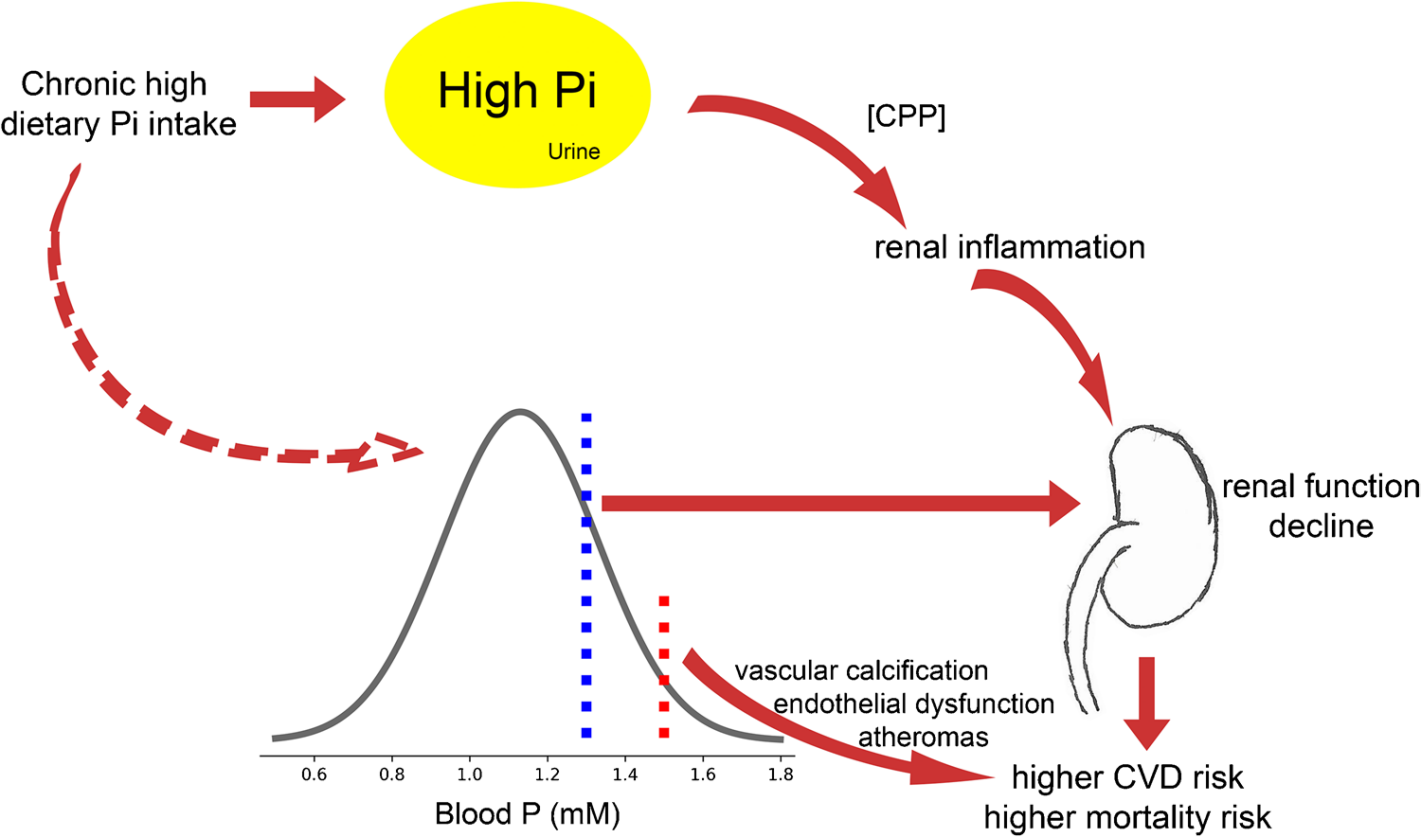
Impact of a chronic high phosphate diet on renal function and associated risks. A chronic high dietary phosphate intake provokes phosphaturia, which leads to the accumulation of calcium phosphate particles and renal inflammation. This further leads to decline of kidney function. A chronic high phosphate intake may also lead to higher phosphate levels than are associated with kidney function decline at values higher than 1.3 mM. Both kidney function decline and hyperphosphatemia (> 1.5. mM) lead to a higher CVD and mortality risk

In summary, although the data are not totally consistent, chronic ingestion of a high phosphate diet over prolonged periods may induce hyperphosphatemia both in human subjects and mice with normal renal function (Fig. 2), despite increases in FGF23 and PTH and a decrease in calcitriol in the blood. Whether the increase in calcitriol levels observed in mice can be explained by hyperparathyroidism needs further clarification. The interesting question whether the chronic increase of PTH and FGF23 may induce renal resistance to their phosphaturic effect also needs further evaluation. In addition, chronic ingestion of putative diet-specific factors that determine whether hyperphosphatemia results in humans need further clarification.

## Impact of high phosphate levels on kidney function

The price for renal elimination of chronic high phosphate intake may be phosphaturia-induced chronic renal injury. Hyperphosphaturia was recently shown to lead to the production of intraluminal calcium phosphate particles. These particles are thought to initiate tubule-interstitial inflammation, via activation of toll-like receptor 4, TLR4, tubule damage, and finally initiation and progression of renal injury [96] (Fig. 2). Indeed, several epidemiological studies have associated high or even normal high phosphate levels in the general population with a higher risk of developing chronic kidney disease (CKD), progressing CKD, and developing end-stage renal disease (ESRD). In a very large retrospective longitudinal cohort study (94,989 subjects) with 11-year observation period, subjects with normal high and high phosphate serum levels (1.26–1.84 mM) had a greater risk for ESRD and mortality [97]. Analysis of subjects of the NHANES III cohort (13,372 subjects with an average follow-up of 9 years) also found an association for higher risk of developing ERSD in subjects with phosphate serum levels higher than 1.3 mM [82]. In a sub-cohort of the Framingham Offspring Study (2275 subjects with a median follow-up of 25.1 years), subjects with phosphate serum levels higher than 1.3 mM were at higher risk to develop CKD [82], yet when mice were fed over 1 year a twofold higher phosphate than in the standard diet, no apparent alteration of kidney function was observed [107]. Of note, the mice were fed a high inorganic phosphate diet but without any concomitant increase of proteins, sodium, or other nutrients that usually are consumed in high phosphate diets in humans.

Several factors affect kidney function, like age and sex. Kidney function, as assessed by eGFR, declines with age [41]. Epidemiological studies suggest that also in aged individuals, higher normal phosphate levels in plasma are associated with a higher mortality rate, accelerated kidney dysfunction, and higher risk of cardiovascular disease [91]. Serum phosphate levels in postmenopausal women are significantly higher than in age-matched men [21], and CKD is more prevalent in women than in men [22]. Further studies are needed to understand these age and sex differences.

Taken together, association studies indicate that phosphate levels over 1.3 mM compromise kidney function (Fig. 2). In addition, experimental and epidemiological evidence suggest that the price for efficient renal elimination of chronic increased and acute phosphate intake may be tubulointerstitial inflammation. This pathogenetic mechanism may constitute a vicious cycle in pre-existing kidney disease of other aetiology or even phosphaturia-induced tubulointerstitial injury itself. With decreasing nephron number, the phosphate excreted per nephron is increasing [99]; more calcium phosphate particles could be built and ultimately might lead to more and progressive renal damage.

## Associated risks of higher phosphate intake and/or blood levels

CKD is a non-communicable disease caused most frequently by diabetes and hypertension. Irreversible renal tissue and functional alterations can lead to progressive loss of kidney function [111]. Worldwide, around 10% of adults are affected by different forms of CKD [50]. Besides impaired kidney function, CKD patients have a higher mortality and cardiovascular disease risk and a diminished quality of life [84]. In CKD patients, FGF23 and PTH increase. In a population of individuals with preserved renal function, PTH increased continuously with decreasing eGFR (starting at or below 126 ml/min), while FGF-23 started to increase, and calcitriol started to decrease below an apparent eGFR threshold of around 100 ml/min [26]. Therefore, these phosphate-sensitive hormones regulating renal phosphate excretion are alterated much earlier i.e., at much higher eGFR values than previously anticipated. Thus, it is likely that this homeostatic effect occurs or is needed to maintain normophosphatemia in stages of renal injury (or even in normal renal function) that are not yet recognized by routine eGFR measurements clinically. The changes in FGF23, PTH, and calcitriol are progressively and near exponentially accentuated with decreasing eGFR along the different stages of CKD. The fact that hyperphosphatemia, detectable in the routine clinical laboratory measurements, is a distinctly late event in progressive CKD [57] documents the high efficiency of this homeostatic response.

### Cardiovascular risk and mortality

Reduced kidney function, CKD, and ESRD increase the risk of cardiovascular disease events and mortality [35, 84]. These associations have been observed in both CKD patients and the general population and discussed extensively [20, 26, 52].

Hyperphosphatemia increases the risk of cardiovascular disease in CKD patients and the general population. The underlying mechanisms are still under investigation, but several studies indicate that phosphate acts directly on arterial vessels by promoting vascular calcification and endothelial dysfunction [108]. The causes of vascular calcification in hyperphosphatemia are several, such as remodelling of vascular smooth muscle cells (VSMC) to chondrocytes or osteoblast-like cells or physico-chemical properties, and those are exacerbated in states of CKD [72]. Additionally, previous studies showed that phosphate directly provokes a concentration-dependent vasoconstrictor effect in aortic rings impairing endothelium-dependent relaxation and induces vascular remodelling and stiffness [27, 98]. Hyperphosphatemia also reduces VSMC proliferation, a risk factor for developing cardiovascular disease, by inducing apoptosis and cell cycle arrest [88]. In addition, phosphate was recently shown to increase de novo cholesterol synthesis in VSMC and macrophages through stimulation of the 3-hydroxy-3-methylglutaryl coenzyme A reductase [113]. Thus, phosphate could also promote CKD-associated atherogenesis by stimulating vascular cholesterol synthesis.

In addition to higher phosphate levels per se, higher levels of FGF23 or lower levels (due to CKD) of the vasculoprotective αKlotho may contribute to a higher incidence of cardiovascular disease in CKD patients, by acting on VSMC and provoking vascular calcifications [98], and by inducing left ventricular hypertrophy [7, 30, 86]. Chronic high FGF-23 levels in humans with X-linked hypophosphatemia and two murine model of this genetic disease were, however, not associated with increased cardiovascular risk [7]. Thus, the relative contribution to the cardiovascular disease burden of progressive CKD per se, hyperphosphatemia, increased FGF-23 and decreased αKlotho levels, and changes in PTH and 1,25(OH)2D need detailed clarification.

### Hypertension

Patients with hypertension are at higher risk to develop CKD [111]. Whereas association studies rather suggest an inverse correlation between high phosphate consumption and risk of hypertension, intervention studies and animal studies suggest the opposite [91]. Twenty healthy young adults ingesting a high neutral sodium phosphate diet for 6 weeks showed elevated phosphate, PTH, FGF23, and αKlotho levels. In addition, mean 24 h increase in systolic and diastolic blood pressure and pulse rate increased significantly [73]. One potential mechanism might be phosphate-induced (directly or indirectly) stimulation of sympatho-adrenergic activity. Further studies suggest that the type of phosphate is crucial and only added phosphate to the diet (inorganic), and not phosphate from animal or plant origin leads to changes in blood pressure [71]. Of note, higher soluble αKlotho have been recently associated with a modest but lower incidence of incipient hypertension in an aged cohort, independently of phosphate, calcium, PTH, FGF23, and calcitriol levels [28].

### Skeletal abnormalities

As mentioned, a high phosphate diet increases not only phosphate levels in plasma but also PTH levels. Animal studies and some human studies indicate that a high phosphate diet is linked to higher bone resorption and lower bone density mass [110]. An intervention study with 11 healthy volunteers showed that increased phosphate consumption as phosphate-based food additive significantly increased osteopontin and osteocalcin concentrations and decreased sclerostin concentrations leading to reduced bone mass density in mice [36]. Mice fed a high inorganic phosphate diet over 12 months showed a marked reduction of bone mass in large part due to bone resorption, which may be caused by increased PTH levels. In addition, subtle chronic acid loads may have contributed to the bone phenotype in this model [107]. Taken together, higher phosphate intake can lead to reduced bone mass.

## Nutritional recommendations for CKD patients

Like for other non-communicable diseases, the so-called unhealthy diets, i.e. high salt and sugar consumption and low intake of vegetables, fruits, and whole grains, increase the risk and worsen the progression of CKD [64]. Dietary adjustments are usually needed in CKD patients as the disease causes metabolic alterations and alters appetite [48]. Restricted protein and sodium intake are recommended for preventing CKD incidence and progression [51], yet the mechanisms and whether high protein diets affect renal function in the general and CKD are still not fully understood [56]. High protein consumption leads first to hyperfiltration and albuminuria, which eventually causes renal function decline. Protein and phosphate consumption are linked, as 84% of phosphate intake is explained by protein intake [49]. Therefore, a protein-restricted diet leads also to decreased phosphate consumption.

Kidney function decline leads to phosphate retention, although higher phosphate levels in plasma are only measured in the later stages of CKD [45, 57, 75]. For stages 3 to 4, it is recommended to restrict phosphate consumption and especially the intake of UPFs. In CKD stage 5, despite offering the potential to reduce phosphate loads, protein restriction is not recommended due to protein wasting [48]. As FGF23 levels increase in the early stages of CKD, it may be recommendable to start nutritional management in CKD patients when altered FGF23 levels are observed. However, FGF23 is not routinely measured in clinical practice. Although further investigations are needed, plant-based diets are becoming popular among clinicians and dietitians treating CKD patients [47]. Targeting the active intestinal phosphate transporters is a promising therapy to control hyperphosphatemia in CKD [23], as low phosphate diet induces upregulation of the intestinal NaPi-IIb transporter and, thereby, leads to higher fractional absorption of phosphate.

## Conclusion

An important proportion of the population is consuming regularly twice the amount of phosphate recommended. Studies indicate that this high phosphate consumption may lead to higher incidence of kidney disease and associated risks such as cardiovascular disease and bone disorders and a higher mortality rate. This chronic high phosphate consumption leads to hyperphosphaturia, which has been recently shown to induce renal inflammation with a consecutive decline in kidney function by accumulation of calcium phosphate particles. This may then lead to a decline in renal phosphate excretion and concomitant development of hyperphosphatemia which leads to further decline of kidney function (via increased calcium-phosphate particles in the tubular lumen of surviving nephrons) and associated risks such as cardiovascular events and higher mortality.
